# Solar panel surface dirt detection and removal based on arduino color recognition

**DOI:** 10.1016/j.mex.2022.101967

**Published:** 2022-12-13

**Authors:** Benjamin O. Olorunfemi, Nnamdi I. Nwulu, Omolola A. Ogbolumani

**Affiliations:** Center for Cyber-Physical Food, Energy and Water Systems (CCP-FEWS), University of Johannesburg, Auckland Park 2006, South Africa

**Keywords:** Color sensor, Dirt removal, Efficiency, Image recognition, Microcontroller, Monitoring, Renewable energy, Solar energy, *Identification of color variation of solar panels*

## Abstract

Color sensing is a technique for identifying physical changes in materials based on appearance assessment. Dirt deposition on solar panels can change their physical appearance and performance. Considering that dirt accumulation on solar panels needs monitoring to make efficient cleaning schedules, reduce unnecessary costs, and optimize solar panel output generation. Color sensing can achieve fast, accurate, and economical dirt detection, unlike the use of robotic cameras, mathematical formulae, and considering varying output current and voltage methods. Here, we introduce a method that detects and removes dirt on solar panels based on TCS3200 and Arduino Uno components. The approach targets (i.) Panel color measurement, calibration, threshold selection process, (ii.) comparison of color measurement values, and (iii.) align further calibration in response to discoloration of solar panels. This method aims to correct the dirt detection methods previously in use. Hence, a high-speed rolling brush arrangement is designed to improve the cleaning of the solar panel without using water. Further investigations of the panel's color may require some improvement in terms of increasing the sensitivity of the color sensor even with increased distance from the solar panel. Combining multiple color sensors may also be necessary.

## Graphical abstract

**Figure:**
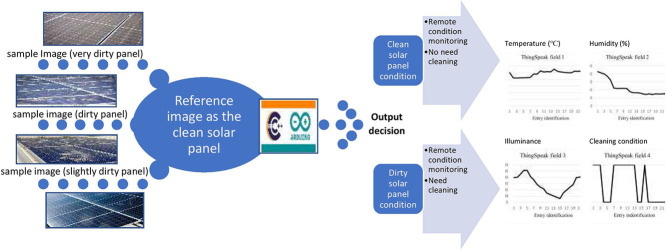

Specifications table

**Table:** 

Subject Area:	Engineering
More specific subject area:	*Solar panel surface cleaning*
Method name:	*Identification of color variation of solar panels*
Name and reference of the original method:	*Automated unsupervised change detection technique from RGB color image.* *M Gomaa* et al. *2019*[1]*.*
Resource availability:	https://www.arduino.cc/en/software*,*https://www.labcenter.com/simulation/*,*https://www.labcenter.com/simulation/*,*

## Method details

### Background

Solar energy is a great alternative energy source for generating electricity because it is renewable and emits no waste [2]. As photovoltaic technology advances, conservation becomes a priority to decrease electricity costs since it requires only the sun's rays for its fuel [3]. Dirt on solar panels' exteriors limits the reception of the sun's energy, causing a significant reduction in electricity produced by the solar panel system [4,5]. The reduction in the solar panels’ output also results in a decrease in the system efficiency while the module degradation is increased. The level of efficiency being recorded when sunlight is converted into electricity is fundamental and this has led to an increased desire for more photovoltaic system developments [6,7]. According to [8], the annual power loss triggered by dust deposition habitually ranges from 5% to 30%. At solar panel installation sites, dirt deposition is recurrent and unpredictable, which makes controlling the dirt deposition rate difficult [9]. The dependence on natural cleaning agents such as snow, wind, and rainfall is unreliable and less effective. Furthermore, removing dirt deposits on solar panels through unplanned maintenance causes significant monetary losses [10]. Therefore, the continual solar panel dust status check is significant to ensure the most excellent power generation [4].

Consequently, it is imperative to investigate the causes of dust movement onto the solar panel surface. The result of the experiment in [11] indicates that the thermophoretic effect contributes to dust deposition on the solar panel. Forces on dust particles include capillary force, electrostatic force, van der Waals force, and gravity, affecting dust immersion rates [12,13]. Also, a humid environment increases forces that cause the stickiness of dust.

A crude method for dirt detection on the solar panel is physical observation by professionals. This method is time-consuming, and it is financially expensive to have technical personnel to regularly observe a giant farm. The cleaning time is a trade-off between the cleaning cost and the acceptable dirt condition for the solar module's efficiency because continuous cleaning causes needless budgets. Solar panels are mostly found on rooftops and in remote fields, so automated dirt recognition and cleaning using robotic systems have proven to be more effective than other methods for detecting dirt accumulation. A malfunctioning dirt detector will result in wrong notification; thereby resulting in a request for panel cleaning action at a needless time, and at a vital cleaning state, the detector may fail.

Many mechanisms have been adopted to bridge the gap between cleaning costs and the fair dirt condition for the efficiency of solar panels [14]. Relatively, to determine whether the solar panel has dust present on it, some studies have been carried out to measure the particle mass of a sample glass or the light transmittance loss [15]. An alternative dirt detection method in [16] calculates the ideal cleaning operation range in days to drop the cleaning budget and maximize earnings from dirty panels by applying a mathematical formula. In the formula, the decided cleaning steps are grounded on the atmospheric settings and the cost of the washing progression. However, calculating the variables is endless, delayed, and leads to irregularity. Hence, the need to automate dirt detection [17].

The dirt detection techniques described in [18,19], assess the drop in solar output power or the short-circuit current caused by dirt on photovoltaic modules; these techniques continuously screen the power output to detect the dirt and activate the essential cleaning process. The current and voltage sensors connected with a controller compute the PV panel output in actual timing and then process the accumulated dust effects on the output power. An advanced approach can steadily deliver satisfactory detection regardless of panel output because the power output is liable to other losses.

In a better approach, theoretical work has proved the possible benefits of using imaging techniques to monitor the exterior characteristics of objects’ conditions. The presently existing imaging techniques adopt optical components, cameras, and software to monitor objects’ surfaces [20]. Withal, this dirt detection technique is complicated and has high costs. However, using flying robots and drones with high-resolution cameras has proved to be a more effective technique, they can capture images in more detail [21]. There also exist errors from the image processing method, which requires advanced methods to transform the images [22].

The status of color for identifying and estimating the physical qualities of materials is featured in [23,24]. Image processing and object detection are achieved in industries using color characteristics while color sensors detect and sort materials according to their color [24]. Also, the environments can be sensed and checked based on the gradations of color sensitivity. This approach can detect changes spontaneously and quickly [1]. They evaluate reflected light in the red, green, and blue (the three colors of white light) scale with corresponding wavelength output. Experimental projects by [25,26], distinguished the physical appearances of strawberry fruit and tomatoes using a color sensor to determine their maturity, organizing, and separating them by considering their color. There is a different value for RGB for every stage of ripening. The object's color signature is an appropriate description for spotting changes in the RGB images. Input images are correlated to find the regions of changing colors, and the contents of the images are identified to match the function, objective, or precision of measurements required.

Color sensors are photoelectric that generate light and detect mirrored light reflected by an object. In [20], a driven rail system carried out dynamic measurements on anthropogenic backgrounds using the TCS3200 color sensor. The sensor was set at the stated elevations in the experiment and traveled the 150 cm line at a steady forward speed. The TCS3200 is equipped with white Light Emitting Diodes (LEDs) to light and illuminate the object's surface for color detection, and the object reflects light to the sensor to determine color intensities. Photodiodes 8 × 8 in TCS3200 are veiled with red, green, blue, (RGB), and transparent optical filters to convert photons into electronic signals [28,29]. TCS3200 can detect the color of the light incident and output square waves with 50% duty cycles. The current-to-frequency converter translated the reflected light intensity into a frequency that can be interfaced with a microcontroller utilizing the digital input and output pins. Compared to other scientific instruments, this sensor is reasonably at a low cost.

The optical properties of solar panels can change due to discoloration. The discoloration can be permanent discoloration due to intense climates or temporary due to the dirt condition of the surface [30]. The permanent discoloration depends on the type of encapsulant used, ultraviolet light, module temperature, humidity, or permeable oxygen [31]. The saturation and reflection losses on incident energy of solar panels are evident in their discoloration. Encapsulant discoloration is the most visible among the degradation modes [32,33]. Brown and yellow pigment on panels develop due to Ethyl Vinyl Acetate (EVA)**,** a result of an uncontrollable chemical reaction from materials within the panel. Several additives used to prevent panel discoloration and strengthen ultraviolet tolerance can disappear over time, causing EVA-based modules to be yellow or brown. The discoloration is extreme in the hot spot areas or damaged cells. It is usually nonuniform and follows scattered patterns. Although innovative approaches reduce discoloration rates, they do not eliminate them [34]. Since the color features of deposited dirt materials can be used to classify clean and unclean solar panels, this paper proposes a more realistic and cost-effective solar panel cleaning technique.

## Design and construction of the dirt detection and cleaning system

The system is made up of hardware and software components. Hardware refers to the physical components, with the microcontroller as the central element of the entire system and the software components are the operating systems that make the system work harmoniously to ensure that the solar panels are always clean, these components are listed in Table 1. Four sensors, two actuators, and network transmission component devices are selected for the implementation. The DHT22 was used as the temperature and humidity sensing component. The Light-dependent resistor (LDR) functions as the illuminance sensor. TCS3200 was used as the color sensor. The robot's movement was controlled using the limit switch sensor. The L298N motor driver function to drive the DC motors. and SIM900 GSM module was used as the network transmission component to wirelessly send the monitored solar panel's parameter and cleaning time data to the Thingspeak Web Server (TWS).

**Table 1:** List of components.

No.	Component
1.	Arduino uno rev3 (ATmega328P microcontroller, 5 V Operating voltage)
2.	LDR Light Dependent Resistor (Maximum voltage 200 V, Peak wavelength 600 nm and Minimum resistance 1.8kΩ)
3.	DHT22 Temperature/Humidity sensor (5 V Operating voltage, Repeatability: Humidity +−1%RH; Temperature +−0.2Celsius)
4.	TCS3200 Color sensor (2.7 *V* − 5.5 V Input voltage, −40oC − 85oC Working temperature)
5.	SIM900 GSM GPRS Module (3.4 − 4.5 V DC, SIM900 Quad-Band – GSM850 / EGSM900 / DCS1800 / PCS1900)
6.	L298N Motor driver (46 V Maximum motor supply, 2A Maximum motor supply, 5 V Logic Voltage, 25 W Maximum power)
7.	DC motor (Gear motor 12 V, 4000 revolution per minute)
8.	Limit switch (3A 125VAC)
9.	Solar panel (30 W Maximum power, 21.8 V Open-circuit voltage, 1.82A Short-circuit current)
10.	Charge controller (10A Maximum current, 6–60 V Input voltage)
11.	Rechargeable battery (12.8 V Output voltage, 7AH Energy charge)
12.	Voltage regulator (1,5 − 35 V Output voltage, 3A Output current)
13.	Roller brush (50 cm Length, 7 cm Diameter)
14.	Polyvinyl chloride casing (60 cm x 20 cm x 10 cm)

The Arduino IDE software is used to write, compile, and upload the sketch codes for the system's function. These codes are written in the C++ programming language. Dirt detection by color sensor ascertains the panel color and compares it with the measured color. Dirt is detected by the system through five main processes: color measurement, color calibration, threshold selection process, comparison of measured result with reference, and setting new reference calibration.

**Fig. 1:**
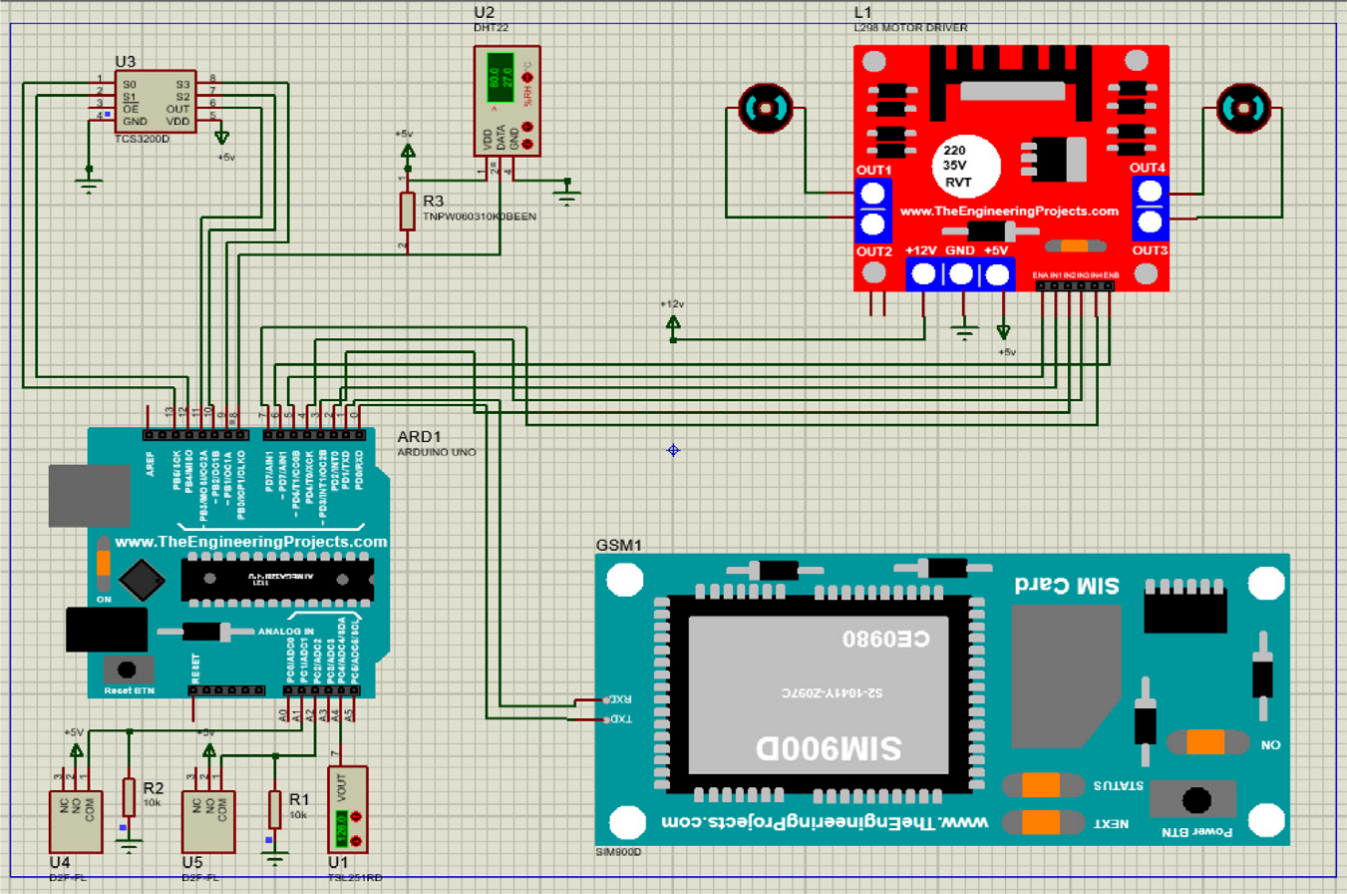
Simulation of the components using Proteus 8 software.

The first step is to connect the components to the microcontroller. The Arduino Uno R3 microcontroller module controls the whole process. Fig. 1 shows the components simulation using the Proteus 8 software.

**Fig. 2:**
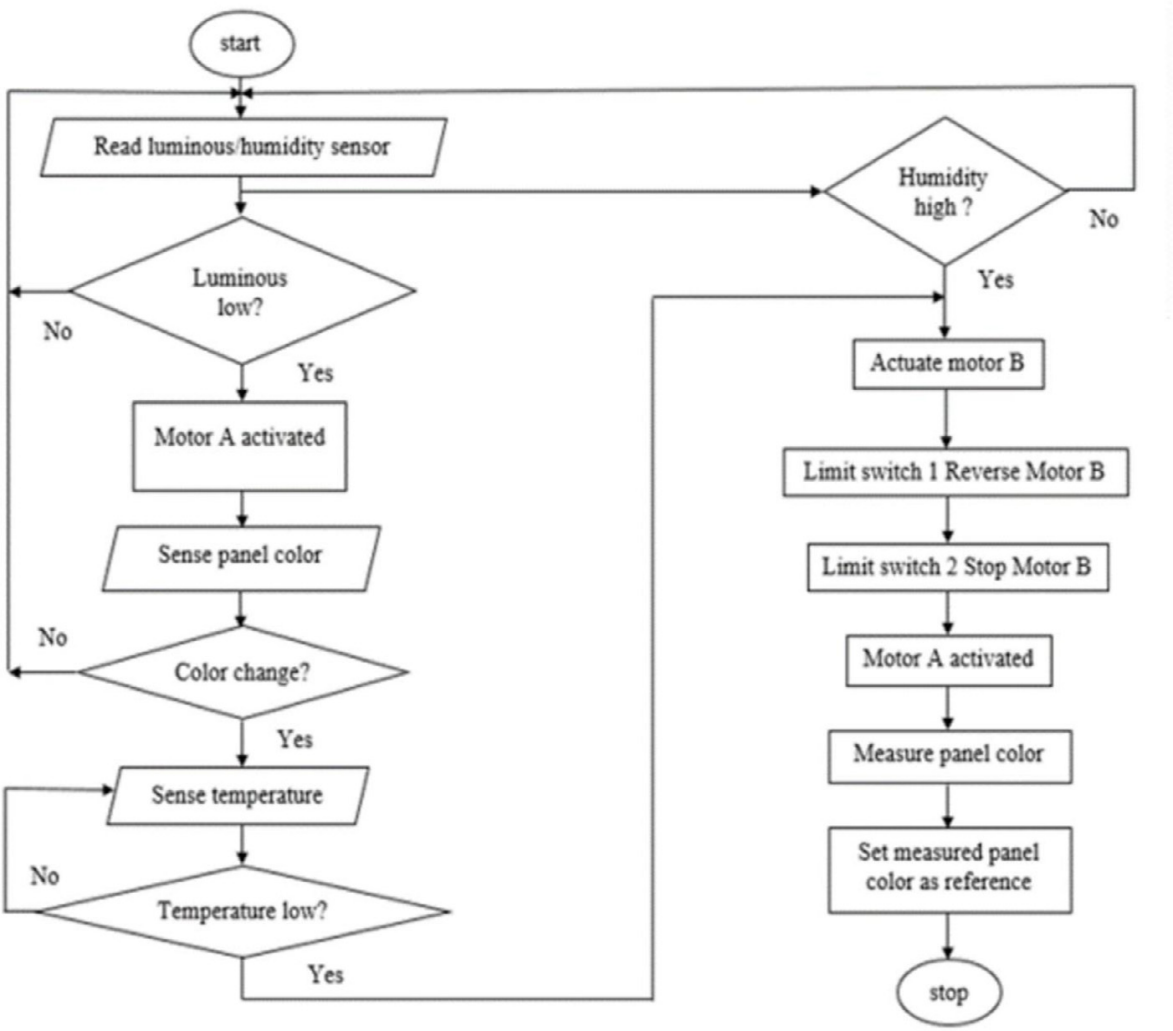
Flowchart of the dirt detection and cleaning process.

Following the system flowchart depicted in Fig. 2, the system is programmed to activate cleaning at two conditions. The first condition involves deviations in the coloration of the photovoltaic cells, and the second involves the measurement of humidity levels. In the solar panel environment, the solar panel temperature and humidity are measured electronically by a digital temperature/humidity sensor. When the controller detects that the humidity threshold level signal has reached 80%, the motor driver will turn on to clean the solar panel. Motor A of the two DC motors in the setup moves the color sensor across the solar panel for color monitoring, while Motor B drives the cleaning robot to remove dirt.

### Calibration and instruction

**Fig. 3:**
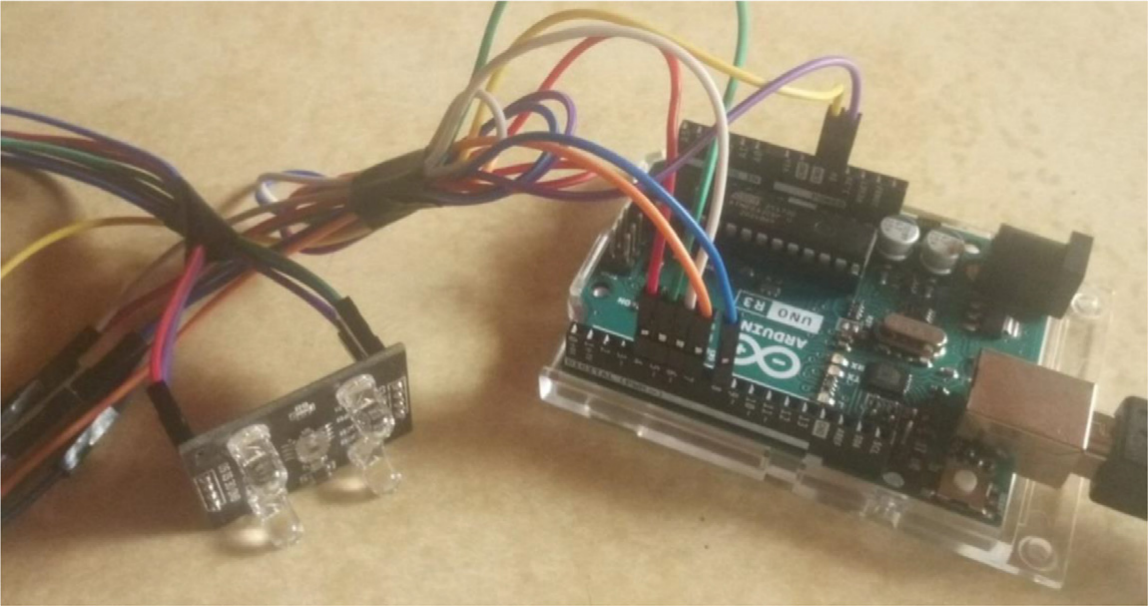
TCS3200 color sensor connection to Arduino Uno Rev3.

The TCS3200 color sensor, as shown in Fig. 3, has eight connection pins linked to the controller module; GND supply ground, VCC supply voltage, OE output enable, OUT frequency output, SO, and S1 for selecting output frequency, and the S2 and S3 for selecting photodiode type.

This section focuses on implementing the instruction code used to detect a change in solar panel color due to surface accumulation of dirt. They are highlighted in the following steps.

**Step 1:** The color processing function controls the logic levels S2 and S3 pins to take the object's colors. The color sensor conveys pulses accepted in this function through the pulse command. The photodiodes connect correspondingly; the S2 and S3 at LOW or HIGH in different orders make selecting photodiodes for a specific color. For Red color intensity, both S2 and S3 pins are set to LOW. S2 to LOW and S3 to HIGH to measure Blue color intensity. Finally, S2 to HIGH and S3 to HIGH measure Green color intensity. Simultaneously, the intensity value is sent to the control system through the converter that produces a square wave. The S0 and S1 pins vary the frequency bandwidth of the output to scaling; either 2%, 20%, or 100%. S0 to LOW and S1 to HIGH effect 2% scaling. S0 to HIGH and S1 to LOW is for 20% scaling and S0 to HIGH and S1 to HIGH affect 100% frequency scale. Scaling the output frequency helps enhance the sensor analyses for different frequency counters or microcontrollers. The map() function denotes the distinguished colors through the RGB model with 0 to 255.

**Step 2:** Calibration is needed to get the correct RGB value of the solar panel. We define the color sensor pins and the three pulse width modulation variables for the Red, Green, and Blue. Pulse width scaling to 20% to match the Arduino. Afterward, set up the serial monitor to read the colors. We can calculate the maximum and minimum disparities in the sensor output frequencies.

The calibration sketch is written to take in the initial sensor data, discourses the color components of the TCS3200 sensor, and conditions the output pin pulse width. The serial monitor displays the output as the color sensor travels over the panel to detect the solar panel color. The following Arduino code accomplishes this:

**Table:** 

#define S0 4
#define S1 5
#define S2 6
#define S3 7
#define sensorOut 8
int redFrequency = 0;
int greenFrequency = 0;
int blueFrequency = 0;
void setup() {
pinMode(S0, OUTPUT);
pinMode(S1, OUTPUT);
pinMode(S2, OUTPUT);
pinMode(S3, OUTPUT);
pinMode(sensorOut, INPUT);
digitalWrite(S0,HIGH);
digitalWrite(S1,LOW);
Serial.begin(9600);
}
void loop() {
digitalWrite(S2,LOW);
digitalWrite(S3,LOW);
redFrequency = pulseIn(sensorOut, LOW);
Serial.print("*R* = ");
Serial.print(redFrequency);
delay(100);
digitalWrite(S2,HIGH);
digitalWrite(S3,HIGH);
greenFrequency = pulseIn(sensorOut, LOW);
Serial.print(" *G* = ");
Serial.print(greenFrequency);
delay(100);
digitalWrite(S2,LOW);
digitalWrite(S3,HIGH);
blueFrequency = pulseIn(sensorOut, LOW);
Serial.print(" *B* = ");
Serial.println(blueFrequency);
delay(100);
}

The sensor calibrates the module measurement by analyzing and recording the frequencies of the reflected light. Categorization of Solar panels is done according to their color values by using calibration. A color sensor test obtains the average value on the sample solar using the TCS3200 sensor, which gives threshold values. TCS3200 color sensor illuminates the material surface with LEDs to sense color light with the help of photodiodes arrays; a photodiode is used in the TCS3200 to detect the colors of incoming objects. The sensor converts the readings from the photodiode into a square wave through the use of a light-to-frequency converter. The photodiode chip can recognize an extensive assortment of colors and output in frequency form, the Arduino input. The experiment uses a convex lens to increase the range of the sensor.

**Step 3:** After detecting the panel's colors, a color sorting system starts and sets the color threshold value; the output frequency's minimum and maximum color variations are calculated, stored, and applied. With the customized threshold, the analysis separates the RGB intensities into ranges of values. Exceeding this value will mean an unbearable dirt condition; that is, a selection range outside of the target range indicates an abnormal image and requires cleaning action, then a command for cleaning is sent. This system sorts colors resulting from the previously calibrated color of the panel and gives an acceptable limit between each change. There are two highlights noted here: the highest and lowest values per RGB component

**Step 4:** An evaluation is achieved between the measured color RGB values and the stored values. According to [1], a mathematical expression is used to analyze the correlation coefficient of the first color input component value compared with the second color input component value. Color change features are produced by detecting the alteration in component values. Eq. (1) is employed to determine the correlation coefficient amongst the initial value f1 and measured value f2 sets.

(1) $$C_{f1f2}=\frac{\sigma_{f1f2}}{\sigma_{f1\;}\times \sigma_{f2}}$$

Where:

*C_f_*_1_*_f_*_2_ = *f*1 and *f*2 correlation coefficient

σ*f*1*f*2 = *f*1 and *f*2 covariance

σ*f*1 = set *f*1 standard deviation

σ*f*2 = set *f*2 standard deviation

The correlation coefficient quantifies the quality of similarity between the two colors of the solar panel (calibrated and measured). The similarity order is either percentage high, Medium, or low. Therefore, the alteration discovery procedure can accurately sense color deviations between two colors.

**Step 5:** Variables are implemented on the final Arduino sketch code to set the panel's color threshold value after cleaning to reflect the solar panel change due to discoloration. In programming, variables keep and term a value that a program will utilize in the next operation. Variable declarations are made by postulating their type and setting them with an initial value (initializing them). The assignment operator (single equal sign) defines variables once declared. The last RGB value is declared a variable for the next operation calibration.

### Experimental set - up

The cleaning mechanism of the solar panel proposed is the dry cleaning method which incorporates a roller brush arrangement to be moved over the panel surface powered by the DC Motor B.

**Fig. 4:**
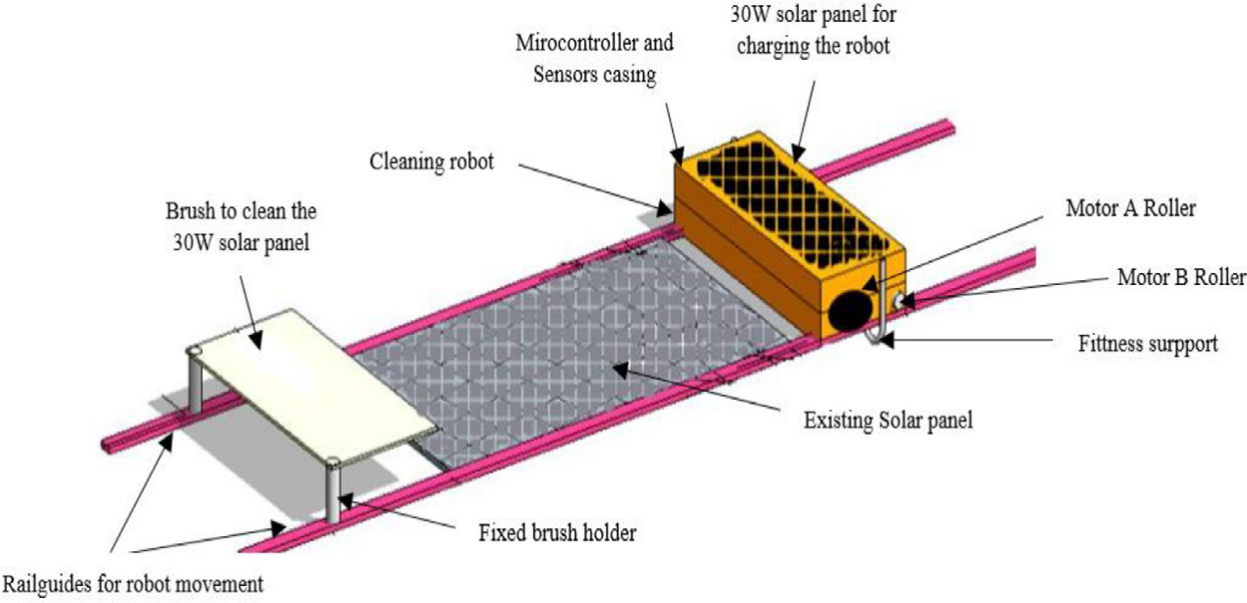
Detailed three-dimensional model of the solar panel cleaning system.

The three-dimensional design of the proposed cleaning robot, drawn using the AutoCAD software, can be seen in Fig. 4. The proposed system, as shown, has two parts, the sensor carrier, which will be moved by motor A and the cleaning mechanism to be moved by motor B. Additionally, the charging solar panel, 30 W (445×510×23 mm dimensions), is dedicated to charging the system battery.

**Fig. 5:**
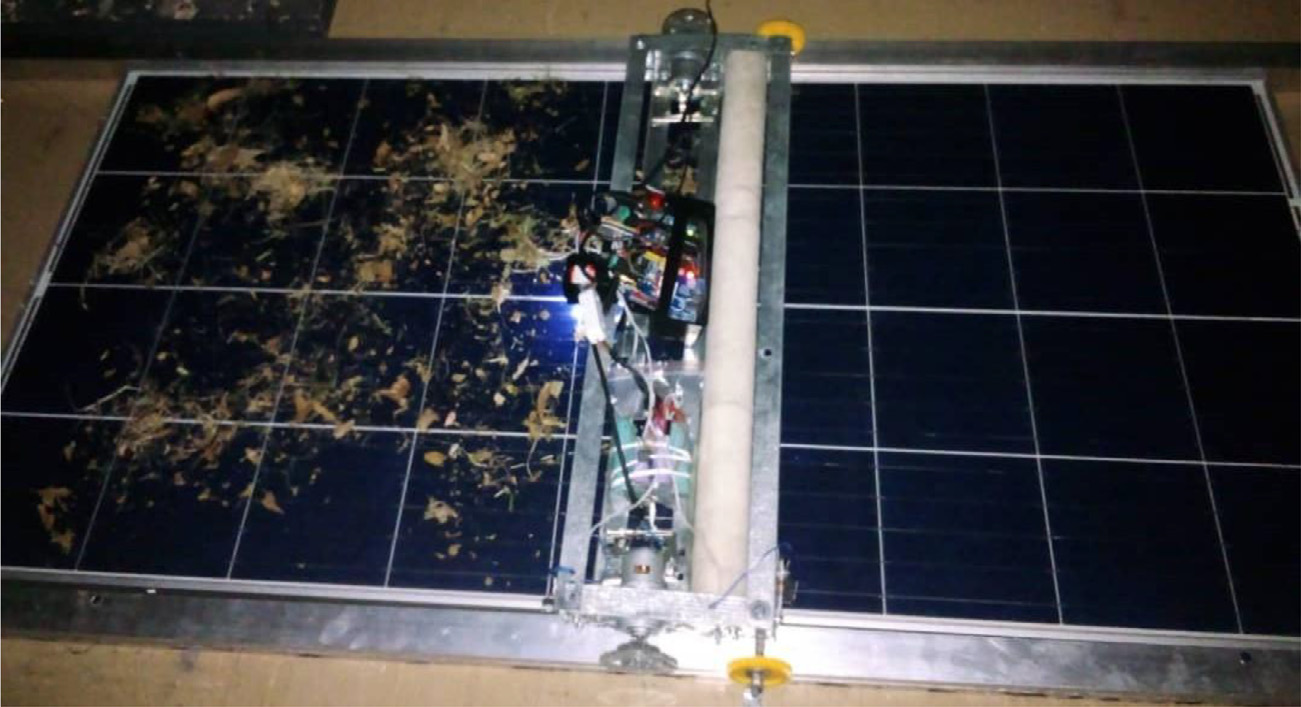
Snapshot of the active solar panel cleaning system.

**Fig. 6:**
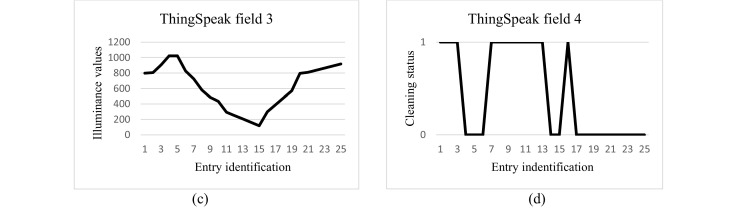
The data visualization on Thingspeak.

The PV panel RGB values at different test conditions are shown in Fig. 6. To produce the reference color of the PV panel, the RGB value of the panel has been measured in a clean state. As can be observed, the RGB variations caused by the dry leaves and animal droppings are small, whereas the RGB value of the dry dust is larger than the reference value. Following the cleaning of the three dirt samples using the designed cleaning system (check Fig. 5 below), the RGB color became normal with the reference RGB color. As seen in the dry dust sample test, the dust completely covers the PV panel. This results in a complete change in the color of the PV panel outside of 0–255. It is important to note that ambient light reflection and the distance of the color sensor from the PV panel may also affect the RGB values.

Embedded in the cleaning system are the motors, the connecting rods, the case shielding the electronic components, and the brush driving systems. The roller brush provides a sweeping motion for cleaning the panel surfaces by spinning at a higher speed than the movement wheels. The cleaning system mechanics was ensured to move along the length of a solar array generating the needed mechanical energy to move the combined mass of the solar panel and overcome the frictional forces to achieve the needed range of motion.

## Method validation

### Remote monitoring test

ThingSpeak analyzes and monitors the data from IoT sensors of the system operation. In this data collection, the temperature data is stored in field 1, the humidity data is stored in field 2, the light intensity chart is stored in field 3, and the cleaning status is stored in field 4 (cleaning On is represented as 1 and cleaning Off is represented as 0). See Fig. 6a, b, c, and d below for the visual representation of the data.

### Solar panel color alteration test

**Fig. 7:**
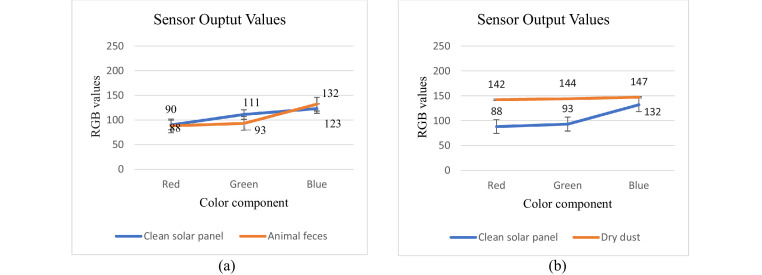
Graph showing the PV color variations when dirt samples are present, (a) animal feces, (b) dry dust.

Fig. 7 illustrates the variation in PV color for different dirt samples. These results illustrate how dirt can alter the color of the PV panel. The RGB variation with animal feces covering a small portion of the solar panel is compared with the variation at the clean solar panel surface; they are similar in Fig. 7(a). There are, however, many differences in RGB values of the dirty sample and the clean solar panel presented in these experiments because every object has its unique characteristics considering the Red, Blue, and Green channels. After measuring the illuminance of the light-dependent resistor (LDR), the test was conducted under dark conditions, and the distance of the sensor from the solar panel was maintained at 3 cm. The code is also edited to cater to the reference color setting.

### Cost evaluation

The cost of cleaning PV systems is largely determined by how frequently they are cleaned and by the economies of removing dirt from them. Studies have shown that automated cleaning of solar panels in regions with less dust instead of with natural methods cannot be economically advantageous [35]. Cleaning cost is calculated by considering capital cost and equipment lifespan and operational expenses (such as electricity, water, and labor).

According to the cost assessment in [36], the Total Yearly Cost of Cleaning per Unit PV Panel for Brush, Microfiber Wiper, Hiring a Cleaning Company, and Intelligent System was 26.42, 1.07, 60, and 1.56 respectively in Euro/Panel/year. An annual cleaning contract that involves hiring a company costs around 60 Euro per panel, followed by cleaning each panel manually with brushes or wipers which cost between 21 and 26 Euro per panel. This smart cleaning system costs 1.5 euros per panel annually, which makes it the most cost-effective solar panel cleaning system. These results demonstrate how combining a few strategies can simplify and reduce costs. Capital and maintenance costs are the major costs associated with the design of cleaning robots in this project. The capital cost of the system after the cumulative cost of the components and devices used amounts to 5000 South African rands equivalent to 277 euros. The estimated number of panels to be cleaned is 20 × 150 W solar panels over a period of 2.5 years. Multiple technologies were integrated into the solar panel monitoring cleaning system as a test field. It has several advantages over other methods of cleaning, including being efficient, intelligent, environmentally friendly, and economically sensible. In one minute, up to 95% of the dust on the PV panel surfaces can be removed by this device.

The system was installed for a 150 W solar panel in the compound of mechanical engineering, University of Johannesburg, Auckland Park, Johannesburg, South Africa. The monitoring and cleaning system was designed and developed to take advantage of the inclination of solar panels to roll the roller brush over the panel for cleaning operation. The components are very durable and low-cost standardized items. Furthermore, the system can be built as a stationary cleaning system for a single array of PV panels and an assembly of adjustable brush lengths to fit the size of the PV panel on site. This is dependent on the applications and the overall system cost.

## Conclusion and further improvements

An inexpensive and passive color sensing on the solar panel that can distinguish the clean and dirty panel surfaces according to their color change has been proposed. The color sensor detects the RGB values of the solar panel. By applying the developed method, an automated solar panel cleaning system can quickly sort the dirt condition according to the color of the panel. The examinations were performed keeping the ambient light low to get accurate RBG values from the color sensor. Obtained results confirm the possibility of the proposed, real-time object classification with a low-cost sensing device that consists of a chip sensor of color that is positioned on the solar panel. As shown in Fig. 6, every object has its unique characteristics and considers the blue (B) green (G), and red (R) channels. Therefore, it is possible to precisely determine what kind of object is observed. Also observed in Fig. 6 was the intersection between the line graph of the clean panel and the dirty panel with animal feces because the animal feces did not cover the whole solar panel, therefore, the color sensor detects the clean areas of the solar panel. It is worth noticing that the panel's color can be accurately recognized at a maximum distance of 3 cm from the TCS3200 color sensor. Disadvantageously, consideration must be given to the distance of the sensor concerning the color being detected. Farther distance reduces the sensor's accuracy. Increased color distortion occurs at a farther distance between the panel and the sensor, and the intensity of the surrounding light. To measure the solar panel's color in the future, different color sensors can be used, or some sensors can be combined. Based on the research results, the threshold values Tr = 88, Tg = 93, and Tb = 132 show the color results of the clean solar panel. Correlation coefficients indicate different degrees of change detection.

It is worth mentioning that the system can be ineffective if it is used to clean old stuck dirt on solar panels such as old bird dropping, and brush elements need to be replaced periodically according to the frequency of use. This concept can execute several functions by monitoring physical variations among objects. The researched project is an improvement over existing systems since it eliminates many of the drawbacks, such as the need for water, manual cleaning, the need for labor. Also, the cleaning frequency and the size of the PV panel's surface area do not have a definitive role in the total cost of the cleaning operation. The surface of the PV panel remains clean always. The system can operate for many years before requiring maintenance. Using the internet of things, the intelligent system can be monitored through the thing speak.

## Declaration of Competing Interest

The authors declare that they have no known competing financial interests or personal relationships that could have appeared to influence the work reported in this paper.

## Data Availability

Data will be made available on request.
